# Determinants of intraoperative radiation during single-level tubular lumbar decompression

**DOI:** 10.1016/j.xnsj.2026.100957

**Published:** 2026-10-08

**Authors:** Sahil Garg, Christopher Franquemont, Patrick Young, Pawan Pathagamage, Arianna K. Gill, Eric Piatt, Justina Lanni, Nivia Garcia, Ben LaCarte, Michael Gallizzi, Sanjitpal S. Gill

**Affiliations:** a Steadman Philippon Research Institute, 181 W Meadow Dr. Suite 1000, Vail, CO, 81657, United States; b The Steadman Clinic, 181 W Meadow Drive, Suite 1000, Vail, CO, United States; c University of Virgnia Orthopaedic Center, 2280 Ivy Road, Charlottesville, VA, 22903, United States

**Keywords:** Tubular decompression, Radiation dose, Fluoroscopy, Body mass index, Automatic exposure control, Minimally invasive spine surgery, Air kerma, Lumbar stenosis

## Abstract

**Background:**

Minimally invasive tubular lumbar decompression requires intraoperative fluoroscopy for retractor docking, yet dosimetry data beyond the learning curve is poorly characterized. Most studies report fluoroscopy time rather than a dose index. We identified patient- and case-level determinants of intraoperative reference air kerma (Kar) during single-level tubular lumbar decompression by a surgeon far into the surgical proficiency learning curve.

**Methods:**

About 114 consecutive patients undergoing single-level unilateral tubular lumbar decompression were retrospectively reviewed between January 2023 and January 2026. The primary outcome was fluoroscopic Kar (mGy). The influence of body mass index (BMI), operative level, age, sex, American Society of Anesthesiologists (ASA) physical status class, laterality, and concurrent discectomy was measured on univariate and multivariable analysis.

**Results:**

Median Kar was 2.13 mGy (interquartile range [IQR] 1.28–3.50) over 4.0 seconds (IQR 3.0–6.0) of fluoroscopy. In the multivariable model BMI was the only independent predictor with a 6.9% increase per unit of BMI (1.069 per kg/m², 95% confidence interval 1.036–1.104, p = .0001). Operative level was not independently significant. Higher BMI was associated with both longer fluoroscopy time (ρ = 0.214, p = .022) and greater Kar per second (ρ = 0.388, p < .001). Secondary analysis suggested higher Kar at upper lumbar levels (L2–L3 and L3–L4) than at lower levels.

**Conclusions:**

BMI is the dominant determinant of intraoperative radiation during tubular lumbar decompression, acting through increased fluoroscopy time and greater C-arm power output rather than surgical complexity. These findings support preoperative radiation planning and dose-reduction strategies targeting automatic exposure control parameters.

## Highlights

• Median reference air kerma was 2.13 mGy over a 4.0-second fluoroscopy time.
• BMI was the only independent predictor: +6.9% air kerma per kg/m² (p = .0001).
• Higher BMI raised dose via both longer fluoroscopy time and more output/second.
• Upper lumbar levels showed higher dose, but not independently of BMI.
• Air kerma is a C-arm dose index, not effective or occupational dose.

## Introduction

Tubular lumbar decompression has emerged as an established alternative to open lumbar decompression. Compared to open techniques, tubular approaches utilize serial dilation and a fixed retractor rather than the broader exposure of a midline incision [1,2]. By limiting paraspinal muscle and posterior soft-tissue disruption, the tubular approach achieves equivalent neural decompression with less associated approach-related morbidity [1,2]. Tubular-based decompression is associated with fewer overall complications, fewer surgical site infections, less blood loss, and shorter length of stay, while operative time, reoperation, dural tear, and long-term patient-reported outcomes do not differ significantly from open decompression [1].

Despite these perioperative advantages, tubular decompression is often associated with fluoroscopy use in order to appropriately dock the retractor system. For this approach, the localization and docking steps of the tubular approach are intrinsic to the operation itself, rather than simply adjuncts for level confirmation such as with open techniques. Modern tubular decompression workflows continue to depend on fluoroscopy to both confirm the target level and to verify trajectory prior to decompression [[2], [3], [4]]. For this reason, the intraoperative radiation associated with tubular decompression remains poorly quantified and has been largely unexamined in contemporary surgical workflows.

Fluoroscopy-dependent spine procedures can expose surgeons to cumulative occupational risk, with potential long-term complications including cataract formation, thyroid cancer, and other malignancies [5]. In a prospective study by Mariscalco et al. [3], radiation exposure at the thyroid/eye, chest, and hand was significantly higher during minimally invasive lumbar microdiscectomy than during open microdiscectomy (1.72 vs. 0.16 mR, 3.08 vs. 0.21 mR, and 4.45 vs. 0.20 mR, respectively) [3]. Cumulative occupational exposure has been associated with a higher prevalence of breast and all-cause cancer among female orthopedic surgeons and with radiation-related skin and nail changes among spine surgeons [6,7]. Intraoperative fluoroscopy has been further linked to cataractogenesis, carcinogenesis, and measurable DNA damage in operating room personnel across multiple surgical specialties, which together show a pattern of cumulative career-long occupational risk [[6], [7], [8], [9]].

Patient-level and case-level determinants of intraoperative fluoroscopy utilization in tubular versus open lumbar decompression remain poorly characterized as well. Patient body habitus has been shown to significantly modify intraoperative radiation emission during minimally invasive spine procedures, and surgeon learning curve has been proposed as an additional modifier, although their specific contributions within dedicated tubular lumbar decompression workflows have not been directly quantified [10]. Operative level may also independently influence total fluoroscopic utilization, yet this has not been quantified for tubular techniques specifically [10,11].

Most existing studies have grouped heterogeneous procedures or evaluated spine surgery broadly as open versus minimally invasive approaches rather than specifically isolating tubular techniques [3,5,11]. As a result, intraoperative fluoroscopy utilization and radiation exposure in single-level tubular decompression, beyond the approach-related learning curve, remain poorly defined. This study aims to characterize intraoperative radiation in the context of patient and case specific determinants during single-level tubular lumbar decompression by a surgeon far beyond the approach-related learning curve. Secondarily, this study aims to assess differences in patient and surgery-related factors of BMI, age, sex, ergonomics (right versus left sided approaches), and operative level results in measurable utilization of radiography.

## Methods

### Study design, setting, and ethics

This retrospective single-surgeon cohort study included consecutive patients who underwent single-level tubular unilateral lumbar decompression at a single hospital between January 2023 and January 2026. All procedures were performed by one fellowship-trained spine surgeon using a tubular retractor system with fluoroscopic guidance for level confirmation and retractor docking. The operating surgeon had performed over 2,000 tubular lumbar decompressions prior to the study period, placing the cohort well beyond the described learning curve for this technique. The WCG Institutional Review Board approved the protocol (IRB No. 1395208) and granted a waiver of informed consent. Reporting follows STROBE guidelines for cohort studies [12].

### Patient selection

Consecutive adult patients who underwent tubular unilateral lumbar decompression during the study period were screened. Eligibility criteria included: (1) age ≥18 years, (2) imaging-confirmed lumbar spinal stenosis, lumbar disc herniation, mild foraminal stenosis, or a combination of these diagnoses (3) failure of nonoperative management, and (4) single-level pathology treated through a unilateral tubular approach. Patients were excluded for multilevel decompression, bilateral decompression in the same sitting, or concurrent instrumented fusion. One patient undergoing decompression for ossification of the ligamentum flavum was excluded as a distinct pathology. These criteria yielded a primary analysis cohort of 114 consecutive patients. Concurrent discectomy was performed in 85 cases (74.6%). This variable was recorded and evaluated as a potential predictor of radiation dose in subsequent univariate and multivariate analysis.

### Surgical technique

All procedures were performed through a tubular retractor system using a muscle-splitting approach. Intraoperative fluoroscopy was used to confirm the target operative level and to guide sequential dilator placement and retractor docking. Once the retractor was secured, decompression was performed under microscopic visualization. Imaging was acquired with a single mobile C-arm (GE Healthcare) operated in pulsed fluoroscopy mode, and the same unit and imaging protocol were used throughout the study period.

### Outcome measures

The primary outcome was the cumulative intraoperative reference air kerma (Kar), recorded directly from the mobile C-arm dose report at the conclusion of each case in milligrays (mGy). Secondary outcomes were cumulative intraoperative fluoroscopy time (seconds) and operative duration (hours). All intraoperative imaging consisted of live pulsed fluoroscopy for level confirmation and retractor docking, supplemented by a small number of saved still images (typically 2–3 per case). The cumulative reference air kerma reported by the C-arm dose report therefore reflects predominantly fluoroscopic output, and its ratio to fluoroscopy time approximates the fluoroscopic dose rate.

### Predictor variables

For each case, the following variables were collected for evaluation as potential predictors of cumulative intraoperative radiation dose: body mass index (BMI), operative level (L2–L3, L3–L4, L4–L5, and L5–S1), patient age, sex, American Society of Anesthesiologists (ASA) physical status classification, laterality (right vs. left), and concurrent discectomy (yes vs. no). The ASA physical status classification grades preoperative physiological health from class I to class VI, with higher classes denoting a greater systemic comorbidity. It was included as a proxy for overall patient health status [13]. All variables were extracted from patient records and verified with the operative note, the intraoperative nursing record, and the C-arm dose report.

### Statistical analysis

All analyses were performed in Python. Continuous variables are summarized as mean ± SD or median (IQR) and categorical variables as counts and percentages. After Shapiro–Wilk testing confirmed non-normality, group comparisons used Mann–Whitney *U* or Kruskal–Wallis tests, and continuous correlations used Spearman’s rank. All tests were 2-sided with α = 0.05. No data were missing for any analysis variable. All analyses were complete-case (n = 114) without imputation.

The primary inferential analysis was a multivariable ordinary least-squares (OLS) regression with the natural log of cumulative reference air kerma (log-Kar) as the dependent variable. This model included all variables (BMI, operative level, age, sex, ASA class, laterality, and concurrent discectomy) and served as the primary analysis, with operative level evaluated by a single overall (omnibus) test. In addition, we pre-specified a secondary hypothesis, based on surgical rationale, that upper lumbar levels (L1–L2 through L3–L4) are more difficult to target than lower levels (L4–L5 and L5–S1) because they lack the lumbosacral junction as an immediate radiographic landmark, and would therefore incur greater radiation. To test this, operative level was dichotomized into upper (L2–L3 and L3–L4) versus lower groups (L4–L5 and L5–S1), and a focused (reduced) model was fit with BMI and this upper-versus-lower grouping. Because the upper-level subgroup was small, this secondary estimate was regarded as underpowered, and its robustness was examined in a post-hoc sensitivity analysis excluding the L2–L3 cases. Model assumptions were assessed by testing residual normality (Shapiro–Wilk) and influence (Cook’s distance). Prior to data collection, the required sample size was informed by a recently published uniportal endoscopic cohort [23], in which higher BMI was independently associated with greater cumulative intraoperative radiation dose. Taking that BMI and log-dose association as the anticipated effect (with an approximate f^2^ = 0.11) using Cohen’s conventions [29], this study required roughly 75 patients to detect the BMI coefficient with 80% power at a 2-sided α of 0.05. The 114 consecutive cases available over the study period exceeded this threshold.

A dose-rate analysis was performed to distinguish whether the BMI and Kar association was driven by increased fluoroscopy duration or by increased C-arm output per unit time. The cohort was divided into BMI tertiles of equal size (n = 38 each), and both cumulative Kar and Kar per second of fluoroscopy (mGy/s) were compared across tertiles. In addition, fluoroscopy time was tested for its association with BMI as a continuous variable (Spearman). Operative duration was added to the reduced regression model as a sensitivity analysis to assess whether it altered the identified BMI coefficient.

## Results

### Patient and case characteristics

Of 115 consecutive cases screened, one was excluded for ossification of the ligamentum flavum, yielding 114 patients. Mean age was 48.9 ± 17.4 years and mean BMI was 25.4 ± 4.2 kg/m². Sixty-two patients (54.4%) were male and 52 patients (45.6%) were female. ASA classification was I in 34 (29.8%), II in 67 (58.8%), and III in 13 (11.4%). Operative levels were L2–L3 (n = 7, 6.1%), L3–L4 (n = 11, 9.6%), L4–L5 (n = 44, 38.6%), and L5–S1 (n = 52, 45.6%). Sixty-seven procedures (58.8%) were left-sided. Concurrent discectomy was performed in 85 cases (74.6%). Patient and case characteristics stratified by operative level are summarized in Table 2.

### Intraoperative outcomes

Median cumulative intraoperative radiation dose (Kar) was 2.13 mGy (IQR 1.28–3.50), median fluoroscopy time was 4.0 seconds (IQR 3.0–6.0), and median operative duration was 0.78 hours (IQR 0.65–0.98). The 75th, 90th, and 95th percentiles of radiation dose were 3.50, 5.47, and 6.13 mGy, respectively.

### Univariable associations with radiation dose (Kar)

BMI was the strongest continuous correlate of cumulative Kar (Spearman ρ = 0.436, p < .0001; Fig. 1). Fluoroscopy time (ρ = 0.673, p < .0001), operative duration (ρ = 0.270, p = .004), and age (ρ = 0.248, p = .008) were also significantly correlated. Among categorical variables, operative level (Kruskal–Wallis p = .007), sex (Mann–Whitney *U* p = .011), and ASA class (Kruskal–Wallis p = .014) were each associated with Kar in univariable testing. Post-hoc pairwise comparisons showed that L2–L3 differed significantly from both L4–L5 (p = .001) and L5–S1 (p = .001), while L3–L4 did not differ significantly from any individual level. Laterality (p = .092) and concurrent discectomy (p = .060) did not reach significance.

**Fig. 1:**
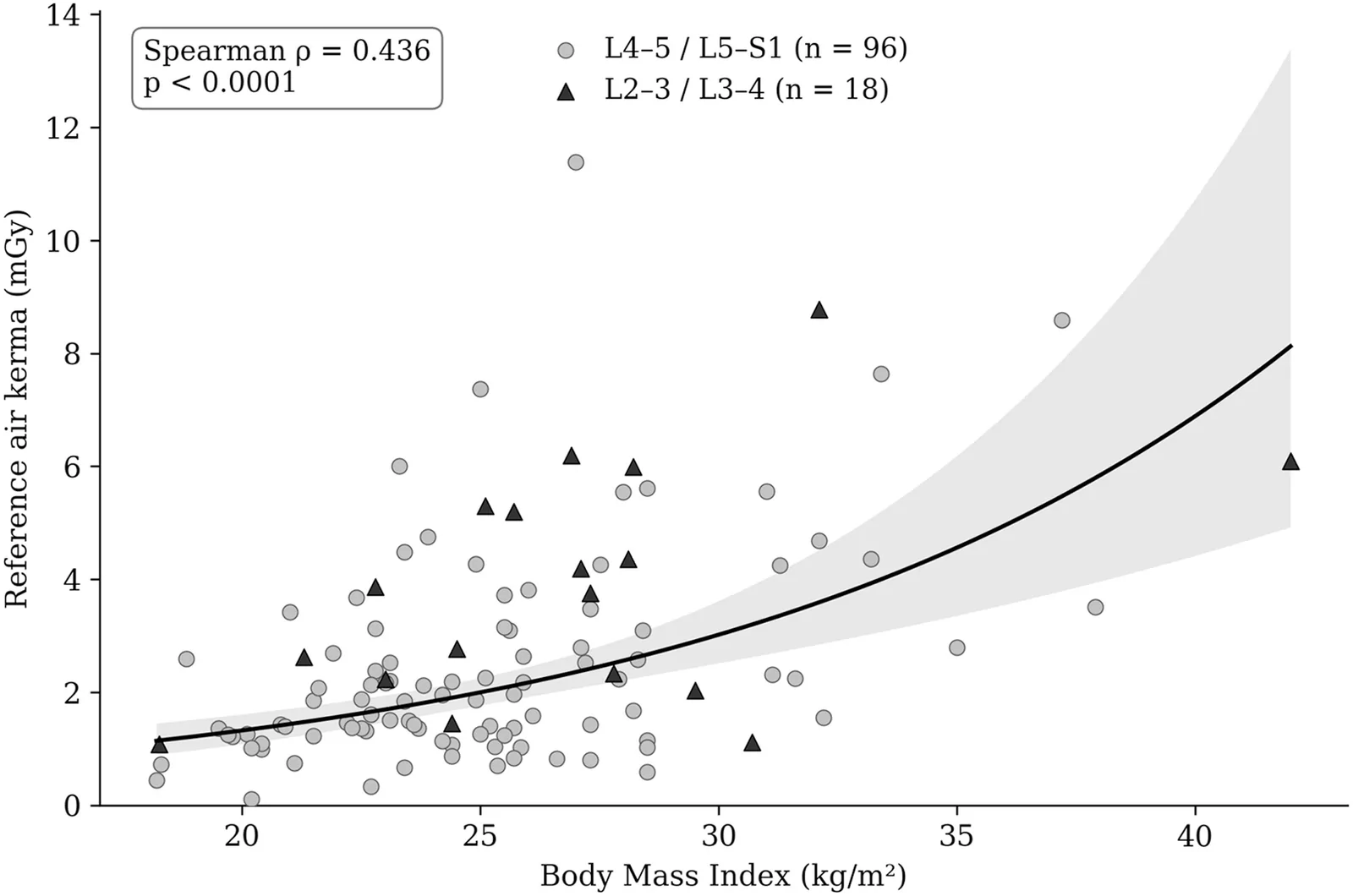
Body mass index (BMI) versus intraoperative reference air kerma (mGy) for 114 tubular lumbar decompressions. Gray circles denote lower levels (L4–L5/L5–S1, n = 96) and black triangles upper levels (L2–L3/L3–L4, n = 18). The solid line is the fitted log-linear regression of air kerma on BMI (back-transformed to the mGy scale) with its 95% confidence band. The bivariate association is summarized by Spearman ρ = 0.436 (p < .0001).

### Multivariable regression

The full multivariable regression on all possible predictors (BMI, age, sex, ASA class, operative level, laterality, and concurrent discectomy) yielded R² = 0.311 (adjusted R² = 0.244, F p < .0001). BMI was the only predictor to reach significance in the full model (exp(β) = 1.069, 95% CI 1.036–1.104, p = .0001), corresponding to a 6.9% increase in cumulative Kar per unit of BMI. Sex, ASA class, age, operative level, laterality, and concurrent discectomy were not independently associated with Kar after adjustment for BMI (Table 3, Fig. 3).

In the full model, the 4 operative levels did not differ significantly overall (p = .30). In the secondary (reduced) model, in which the upper levels (L2–L3 and L3–L4) were pooled and compared with the lower levels (L4–L5 and L5–S1), upper-level cases showed a 50.9% higher Kar (exp [β] = 1.509, 95% CI 1.084–2.100, p = .015). However, in a post-hoc sensitivity analysis excluding the 7 L2–L3 cases, the upper-level effect was attenuated and no longer significant (exp [β] = 1.321, 95% CI 0.875–1.994, p = .18). Although this upper-versus-lower comparison was pre-specified, the post-hoc findings suggest that the estimate is underpowered. This finding should be interpreted with caution pending confirmation in larger cohorts.

### BMI results

To distinguish whether the BMI and Kar association was driven by increased fluoroscopy duration or increased C-arm output per unit time, the cohort was stratified into BMI tertiles of equal size (low 18.2–23.1 kg/m², n = 38, mid 23.3–26.1, n = 38, high 26.6–42.0, n = 38). Equal-size BMI tertiles were chosen over WHO classification cutpoints because the cohort's BMI distribution (mean 25.4 ± 4.2 kg/m²) would have produced severely unbalanced groups under WHO categories, reducing statistical power to detect the dose-rate gradient that is the primary objective of this analysis.

Median cumulative Kar rose monotonically across tertiles: 1.44, 1.91, and 3.29 mGy (Kruskal–Wallis p = .0002; Fig. 2A). Median Kar per second of fluoroscopy also rose progressively: 0.428, 0.533, and 0.681 mGy/s (Kruskal–Wallis p = .0016, Fig. 2B). Fluoroscopy time itself also rose with BMI (Spearman ρ = 0.214, p = .022, median 3.4, 4.0, and 5.0 seconds across the low, mid, and high tertiles, Kruskal–Wallis p = .066), indicating that higher BMI increased Kar through both a longer fluoroscopy time and a greater Kar per second. The Spearman correlation between fluoroscopy time and cumulative Kar strengthened across tertiles (ρ = 0.550, 0.634, and 0.809 for low, mid, and high, respectively, all p < .001), indicating tighter fluoroscopy-to-Kar coupling in higher-BMI patients.

**Fig. 2:**
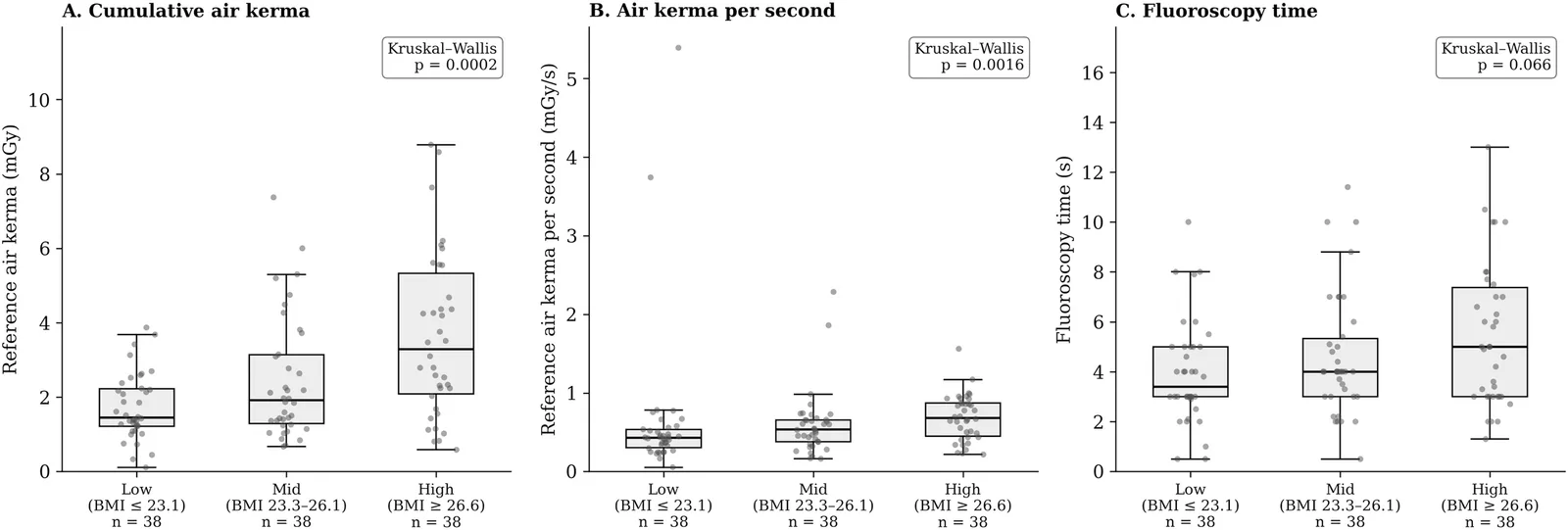
Reference air kerma, air kerma per second, and fluoroscopy time by BMI tertile (n = 38 per tertile with individual cases overlaid). (A) Cumulative air kerma rises across tertiles (Kruskal–Wallis p = .0002). (B) Air kerma per second also rises (p = .0016), consistent with greater C-arm output per unit time in higher-BMI patients. (C) Fluoroscopy time rises across tertiles but does not reach significance on the categorical test (p = .066), though it is significant as a continuous association (Spearman ρ = 0.214, p = .022). Together these show higher BMI raises air kerma through both longer fluoroscopy time and greater output per second.

**Fig. 3:**
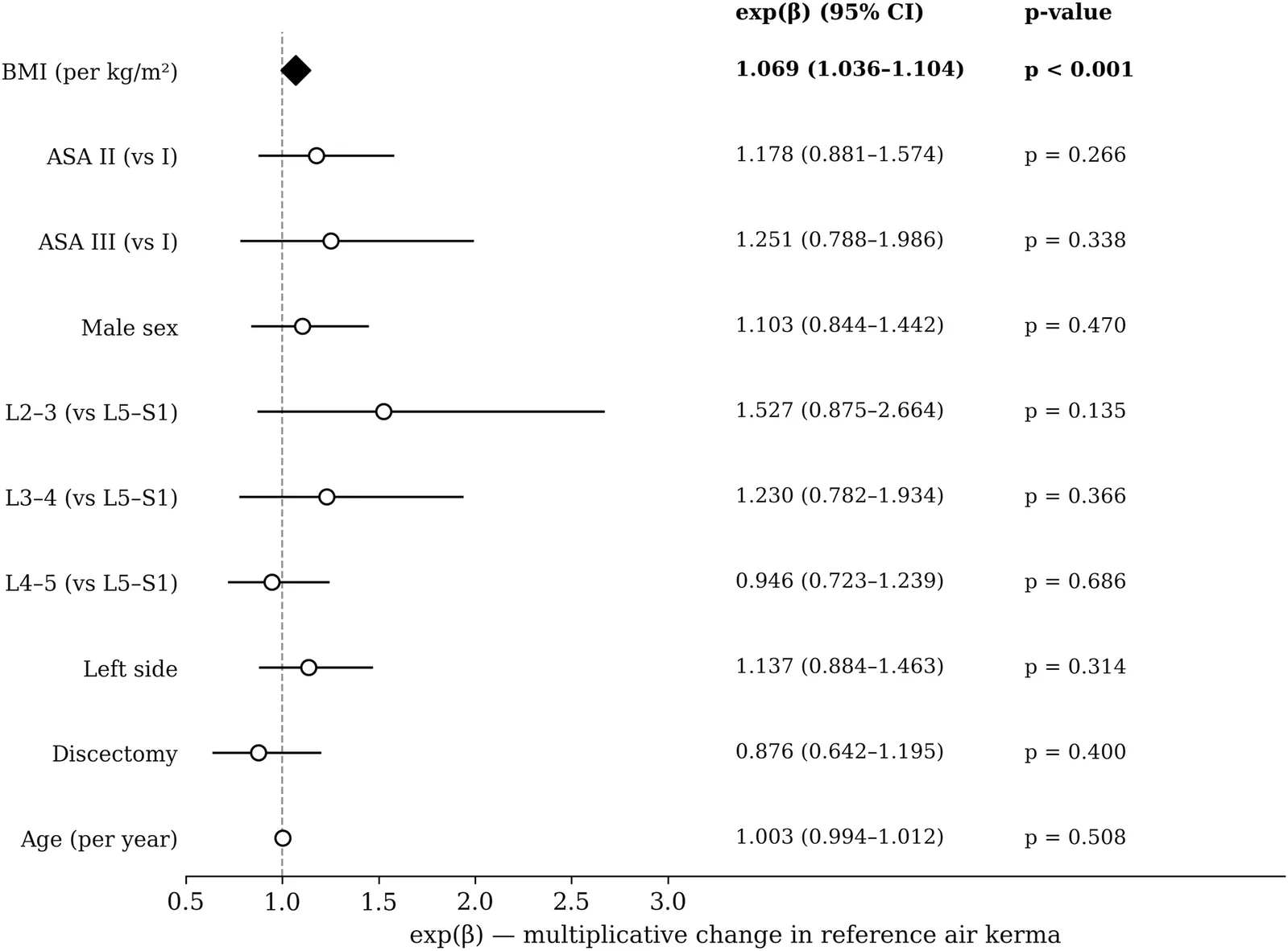
Forest plot of the full multivariable regression (primary analysis) of log-transformed reference air kerma (R² = 0.311). BMI was the only independent predictor (exp [β] = 1.069, p < .001, +6.9% per kg/m²).

BMI was not significantly associated with operative duration (Spearman ρ = 0.161, p = .087). When operative duration was added to the reduced regression model, the BMI coefficient was unchanged (exp [β] = 1.078, p < .0001), and operative duration was not significant (p = .613). The model R² increased negligibly from 0.259 to 0.261. This sensitivity analysis indicates that the BMI and radiation association is independent of operative duration, consistent with greater output per unit time rather than longer or more complex single level decompressive surgery.

### Clinical scenario predictions

In a post-hoc analysis, predicted Kar ranged from 1.28 mGy (BMI 20, lower level) to 8.94 mGy (BMI 40, upper level), a 7.0-fold difference. At lower lumbar levels, predicted Kar (95% CI) was 1.28 (1.05–1.56), 1.88 (1.65–2.14), 2.75 (2.27–3.34), 4.04 (2.95–5.54), and 5.93 (3.78–9.30) mGy for BMI values of 20, 25, 30, 35, and 40 kg/m², respectively. At upper lumbar levels, the corresponding predictions were 1.93 (1.35–2.77), 2.83 (2.09–3.84), 4.15 (3.04–5.68), 6.10 (4.17–8.91), and 8.94 (5.52–14.49) mGy.

## Discussion

### Absolute dose in context

The determinants of intraoperative radiation dose during tubular lumbar decompression are poorly characterized. Existing studies vary in technique and reporting metrics, and none provide a reliable benchmark for surgeons beyond the learning curve [[14], [15], [16], [17]]. The primary outcome in this study is cumulative air kerma (Kar) recorded from the C-arm and reported in milligrays (mGy).

Because reference air kerma reflects radiation output at a defined reference point, it is most appropriately interpreted against the air-kerma levels associated with tissue effects rather than converted to whole-body effective dose. Radiation effects are conventionally divided into deterministic effects, such as skin injury and cataracts, which occur only above dose thresholds, and probabilistic effects, such as cancer, which are modeled without a threshold and accumulate with each exposure [18]. The cumulative Kar in this cohort (median 2.13 mGy and a maximum below 10 mGy) is far below the dose thresholds at which deterministic skin injury occurs, and acute tissue injury is likely not a concern at these doses. While a single procedure appears to create minimal risk to the individual patient, the primary-beam output that Kar reflects is also the source of scatter to the operating team. Small per-case doses compound across the thousands of procedures a high-volume surgeon performs over a career. Occupational dose, however, depends on shielding, positioning, and distance from the source and was not measured in the present study.

The median fluoroscopy time of 4.0 seconds in the present cohort is substantially lower than that reported for comparable fluoroscopically guided lumbar procedures. Lumbar microdiscectomy has been reported to require a mean fluoroscopy time of 10.2 seconds [19], and open lumbar decompression approximately 13.2 seconds [17]. Even a routine lumbar transforaminal epidural steroid injection involves longer fluoroscopy, averaging approximately 0.7 minutes with a mean patient dose of 136.8 mGy, markedly higher than the 2.13 mGy recorded here [20]. The brief fluoroscopy and low cumulative Kar in this cohort therefore reflect economy of fluoroscopy by an experienced surgeon rather than any difference in imaging equipment or technique.

Existing studies report radiation in various metrics, and their distinctions are important for cross-study comparison. Reference air kerma (mGy) measures the energy released by the x-ray beam per unit mass of air at the patient’s skin surface, it reflects what the beam delivers at a single point but does not account for irradiated area or tissue sensitivity. Dose-area product (DAP), reported in mGy·cm² or Gy·cm², multiplies the air kerma by the cross-sectional area of the beam. Because DAP folds in beam area, its numerical values are inherently much larger than the corresponding air kerma, even though no single point received more radiation, as a result, DAP figures cannot be compared directly to air kerma values. Whole-body effective dose (mSv) goes a step further by weighting absorbed dose to each organ by that organ’s radiosensitivity and by the fraction of the body irradiated, producing a single estimate of overall biologic risk. Converting among them requires assumptions about beam geometry, patient size, and organ position, so the 3 metrics are not interchangeable. For a given fluoroscopic exposure, air kerma (mGy) will be numerically smaller than the corresponding DAP (mGy·cm²), which scales with beam area, and generally larger than the whole-body effective dose (mSv), which is attenuated by the small fraction of the body irradiated during a focused procedure such as tubular docking. Our primary outcome is reference air kerma (mGy). Readers should exercise caution when comparing these values to studies reporting DAP or effective dose.

Merter et al. reported dose-area products of 1.01, 1.39, and 2.46 Gy·cm² for microendoscopic discectomy, unilateral biportal endoscopic discectomy, and percutaneous endoscopic lumbar discectomy, respectively [15]. Mulconrey et al. reported fluoroscopy time for open lumbar decompression but did not separate patient dose by procedure type, underscoring the limitations of fluoroscopy time as a proxy for radiation dose [17]. Direct comparison across these studies is limited by heterogeneous reporting metrics (Table 1). Further studies comparing tubular to endoscopic techniques and associated radiation utilization are therefore warranted.

**Table 1:** Literature comparison of intraoperative radiation exposure during minimally invasive lumbar decompression and discectomy.

Study	Journal	Procedure	n	Fluoroscopy time (s)	DAP (Gy·cm²)*	Cumulative air kerma (mGy)*
Kukreja et al. [10]†	J Neurosurg Spine	MIS microdiscectomy/decompression	211	20.67	—	12.62 (SD 2.75)
Merter et al. [14]	World Neurosurg	Microendoscopic discectomy	25	4.6	1.01	—
Merter et al. [14]	World Neurosurg	Unilateral biportal endoscopic discectomy	25	19.3	1.39	—
Merter et al. [14]	World Neurosurg	Percutaneous endoscopic lumbar discectomy	25	34.9	2.46	—
Grelat et al. [18]	World Neurosurg	Lumbar microdiscectomy	62	10.2	0.94	—
Crawley et al. [27]‡	Br J Radiol	Lumbar discectomy	26	6	1.23	—
Current study	—	Tubular lumbar decompression	114	4.0 (3.0–6.0)	—	2.13 (1.28–3.50)

Values are mean or mean ± SD as reported; the current study reports median (interquartile range).

⁎ Dose-area product (DAP) and cumulative air kerma are distinct quantities and are not directly interchangeable.

† Kukreja et al. [10] reported radiation emission (mGy), which was strongly body mass index-dependent, ranging from a median of 6.77 mGy in normal-weight patients to 35.36 mGy in class III obesity.

‡ Crawley et al. [27] reported median values; the discectomy technique was not specified and predates routine minimally invasive or tubular discectomy.

**Table 2:** Patient and case characteristics stratified by operative level.

Variable	L2–L3 (n = 7)	L3–L4 (n = 11)	L4–L5 (n = 44)	L5–S1 (n = 52)	Total (N = 114)	p-value
Age (y), mean ± SD	64.6 ± 14.9	61.4 ± 11.2	50.5 ± 16.0	42.7 ± 17.1	48.9 ± 17.4	**<.001**
BMI (kg/m²), mean ± SD	28.7 ± 6.7	25.8 ± 3.6	24.9 ± 4.4	25.2 ± 3.6	25.4 ± 4.2	.27
Male sex, n (%)	6 (85.7)	7 (63.6)	21 (47.7)	28 (53.8)	62 (54.4)	.27
ASA class, n (%)						.54
I	0 (0.0)	4 (36.4)	14 (31.8)	16 (30.8)	34 (29.8)	
II	6 (85.7)	5 (45.5)	24 (54.5)	32 (61.5)	67 (58.8)	
III	1 (14.3)	2 (18.2)	6 (13.6)	4 (7.7)	13 (11.4)	
Left side, n (%)	6 (85.7)	5 (45.5)	26 (59.1)	30 (57.7)	67 (58.8)	.40
Discectomy, n (%)	4 (57.1)	7 (63.6)	32 (72.7)	42 (80.8)	85 (74.6)	.40
Reference air kerma (mGy), mean ± SD	5.0 ± 2.2	3.1 ± 1.8	2.5 ± 2.2	2.4 ± 1.6	2.6 ± 2.0	**.007**
Median (IQR)	5.2 (3.3–6.0)	2.3 (1.7–4.3)	1.8 (1.2–3.2)	2.0 (1.3–2.9)	2.1 (1.3–3.5)	
Fluoroscopy time (s), mean ± SD	8.6 ± 4.0	4.9 ± 2.6	4.7 ± 2.7	4.2 ± 2.3	4.7 ± 2.8	**.009**
Median (IQR)	7.0 (6.5–9.0)	4.2 (3.4–5.7)	3.9 (3.0–5.6)	4.0 (2.9–5.0)	4.0 (3.0–6.0)	
Operative duration (h), mean ± SD	1.0 ± 0.2	0.9 ± 0.2	0.9 ± 0.3	0.8 ± 0.2	0.8 ± 0.3	**<.001**

ASA, American Society of Anesthesiologists physical status classification; BMI, body mass index; IQR, interquartile range.

Bold p-values indicate statistical significance (p < 0.05).

Continuous variables compared using Kruskal–Wallis test; categorical variables compared using chi-square test or Fisher exact test as appropriate.

**Table 3:** Multivariable linear regression of log-transformed reference air kerma (log-Kar).

Predictor	β	Exp (β)	% Change	95% CI	p-value
Full model (R² = 0.311, adjusted R² = 0.244)					
BMI (per kg/m²)	0.0672	1.069	+6.9	1.036–1.104	**.0001**
ASA II (vs. I)	0.164	1.178	+17.8	0.881–1.574	.266
ASA III (vs. I)	0.224	1.251	+25.1	0.788–1.986	.338
Male sex	0.098	1.103	+10.3	0.844–1.442	.470
L2–L3 (vs. L5–S1)	0.423	1.527	+52.7	0.875–2.664	.135
L3–L4 (vs. L5–S1)	0.207	1.230	+23.0	0.782–1.934	.366
L4–L5 (vs. L5–S1)	−0.055	0.946	−5.4	0.723–1.239	.686
Left side	0.129	1.137	+13.7	0.884–1.463	.314
Discectomy	−0.132	0.876	−12.4	0.642–1.195	.400
Age (per year)	0.003	1.003	+0.3	0.994–1.012	.508
Reduced Model (R² = 0.259, Adjusted R² = 0.246)					
BMI (per kg/m²)	0.0767	1.080	+8.0	1.049–1.111	**<.0001**
Upper level (L2–L3/L3–L4 vs L4–L5/L5–S1)	0.4112	1.509	+50.9	1.084–2.100	**.015**

Dependent variable: natural log of reference air kerma (log-Kar). β, unstandardized regression coefficient; exp (β), exponentiated coefficient representing the multiplicative change in Kar (Dose) per unit change in predictor; % Change, percentage change in Kar (Dose) per unit change in predictor; CI, confidence interval.

The full model, which includes all 10 predictors entered simultaneously, is the primary analysis. The reduced model is a pre-specified focused model testing the a priori hypothesis that upper lumbar levels (L2–L3 and L3–L4) incur greater radiation than lower levels (L4–L5 and L5–S1). The upper-level estimate is underpowered and should be interpreted with caution.

Reference categories: ASA I, L5–S1, female, right side, no discectomy. Bold p-values indicate statistical significance (p < .05).

All 114 cases were performed by a single surgeon beyond the tubular decompression learning curve, with a consistent docking workflow concentrating fluoroscopy at initial dilator placement, and a single C-arm and imaging protocol were used throughout, eliminating equipment-level variability. The 2.13 mGy median is therefore a protocol-controlled benchmark that surgeons adopting tubular decompression can use to guide expectations and examine a developing practice.

### BMI as the dominant patient-level modifier

Kukreja et al. reported that BMI was significantly associated with increased radiation dose across minimally invasive spine procedures, independent of procedure type [10]. Our findings extend this relationship to tubular decompression, where fluoroscopy is concentrated at a single brief docking step. In the reduced model, each additional kg/m² was associated with an approximate 8.0% increase in cumulative dose (p < .001). The full model yielded a concordant estimate of +6.9% per kg/m² (p < .001). A 10-unit BMI increment (eg, 20–30 kg/m²), corresponds to approximately a 2.2-fold increase in predicted dose. In a cohort with a highly standardized operative workflow, this consistency suggests that body habitus is a genuine patient-specific determinant of dose. Our finding is further supported by Safaee et al. [21], who demonstrated a significant correlation between BMI and fluoroscopy-related radiation dose in minimally invasive transforaminal lumbar interbody fusion [21].

This effect warrants particular attention because tubular decompression is increasingly used in heavier patients, including the morbidly obese, due to less tissue damage, smaller incisions, and lower infection rates than open surgery. In a series of 961 MIS posterior lumbar cases performed through tubular dilators, 423 patients had a BMI of 30 or greater, including 106 with morbid obesity (BMI ≥ 40), reflecting the breadth of body habitus across which tubular techniques are now routinely employed [22]. Surgeons who perform high-volume tubular decompression may therefore become more likely to operate on the patient population most susceptible to increased intraoperative radiation exposure, making BMI a clinically relevant modifier of cumulative career-long exposure.

### Higher BMI increases radiation through both longer fluoroscopy and greater output per second

Higher-BMI patients may accumulate greater radiation for 2 reasons: greater soft-tissue density may demand more fluoroscopy time to achieve adequate visualization, and the C-arm may increase output per unit time to compensate for beam attenuation, independent of how long the beam is on. Both were evident in this cohort. Across BMI tertiles, cumulative Kar (p = .0002) and Kar per second of fluoroscopy (p = .0016) rose monotonically (Fig. 2), and fluoroscopy time itself also increased with BMI (ρ = 0.214, p = .022). Higher-BMI patients therefore received both more fluoroscopy and more Kar per second, rather than more of either alone. The Spearman correlation between fluoroscopy time and cumulative Kar strengthened progressively across tertiles (ρ = 0.550, 0.634, and 0.809, all p < .001), indicating tighter fluoroscopy-to-Kar coupling in heavier patients, as each second of exposure contributed a larger increment of Kar.

BMI was not significantly associated with operative duration (ρ = 0.161, p = .087), and adding operative duration to the regression left the BMI coefficient essentially unchanged (p < .001), with operative duration itself non-significant (p = .613). The BMI–radiation association is therefore independent of operative duration and is more consistent with greater output per unit time than with added surgical complexity. This pattern is consistent with automatic exposure control, in which the C-arm increases kilovoltage or tube current to maintain a target signal at the image intensifier as beam attenuation rises, so each second of fluoroscopy delivers more radiation in larger patients [10]. Projection, positioning, magnification, and collimation may also contribute, and the present data do not isolate automatic exposure control as the sole mechanism. In a separate endoscopic decompression cohort performed by a different surgeon with a separate imaging workflow, the per-unit BMI effect on cumulative Kar was +6.7% per kg/m², closely matching the present findings [23]. Despite these 2 techniques sharing little of their surgical workflow and having been performed by 2 different surgeons, there is a nearly identical BMI related dose gradient. Because higher BMI increases radiation through both longer fluoroscopy and greater output per second, dose-reduction efforts should address both: shortening beam-on time (for example, pulsed fluoroscopy and last-image-hold) and lowering dose per image (tight collimation, minimized magnification, and optimized image-intensifier geometry) [5,24].

### Operative level as a case-level modifier

Operative level was not an independent predictor of Kar in the full model, in which the 4 levels did not differ significantly overall (omnibus p = .30). In the prespecified reduced model, pooled upper levels (L2–L3 and L3–L4, n = 18) were associated with a 50.9% higher Kar than lower levels (L4–L5 and L5–S1, n = 96; exp [β] = 1.509, 95% CI 1.084–2.100, p = .015). This estimate was not robust: it was driven largely by the 7 L2–L3 cases and lost significance when they were excluded (p = .18), and the small subgroup produced wide confidence intervals. Although this upper-versus-lower comparison was pre-specified, the estimate was underpowered and not robust, and should therefore be interpreted as preliminary pending confirmation in larger cohorts. Two mechanisms could plausibly contribute if the association is real. At L2–L3 and L3–L4 the lumbosacral junction is unavailable as an immediate radiographic anchor, which may prompt additional confirmatory imaging during docking, [25] and greater soft-tissue thickness at upper segments may increase output per image [10]. For upper-lumbar tubular decompressions, careful preoperative level localization with a single well-placed confirmatory image may help limit radiation [25].

### Clinical scenario range and counseling implications

The reduced-model equation predicts dose across a range of patient and case combinations, with a 7.0-fold worst-to-best ratio (both variables known to the surgeon at the preoperative visit). A nonobese patient at a lower level (BMI 20 with L4–L5 or L5–S1 pathology) has an anticipated Kar of 1.28 mGy, compared with 8.94 mGy for a severely obese patient at a higher level (BMI 40 with an L2–L3 or L3–L4 pathology) undergoing a decompression. Understanding these expectations before surgery gives surgeons a realistic sense of the anticipated exposure and helps them plan dose-reduction strategies before the first image is taken. Of note, these values may be greater for a surgeon early or midway through the learning curve for docking and placing tubular retractor systems.

### Radiation stewardship in tubular lumbar decompression

The patient- and case-level determinants identified in this study can help guide a radiation-stewardship framework for both the patient and the surgical team in tubular spine surgery. Standard radiation stewardship measures for minimally invasive lumbar fluoroscopy, including pulsed fluoroscopy, tight collimation, last-image-hold, minimized magnification, and personal shielding, have been described previously [23].

At the patient level, BMI should be treated as a preoperative flag that prompts heightened stewardship before the case. Because output per image tends to be higher in larger patients, per-image controls (collimation, low magnification, and image-intensifier positioning) are best set before the first exposure rather than adjusted mid-case. Pulsed fluoroscopy has been reported to reduce fluoroscopy time by approximately 80% in minimally invasive lumbar surgery without compromising procedural efficiency, [26] and collimation reduces scatter to the surgeon’s hand and thyroid by approximately 65% compared with continuous fluoroscopy [27]. Because higher BMI increases radiation through both longer fluoroscopy and greater output per image, these measures reduce dose through complementary pathways (shorter beam-on time and lower dose per image).

At the case level, upper-lumbar decompressions may warrant similar preoperative attention, although the upper-level effect was underpowered and should be interpreted with caution. As general practice, a single well-placed preoperative localization image may reduce the number of intraoperative exposures. Combined with the measures described above, these practices offer a practical way to limit intraoperative radiation in tubular spine surgery.

### Concurrent discectomy and other candidate modifiers

Eighty-five of 114 cases (74.6%) included a concurrent discectomy. A borderline univariable association with Kar (median 1.87 vs. 2.79 mGy, p = .060) was observed in a direction opposite to what added surgical complexity would predict and likely reflects age confounding. Patients in the discectomy group were substantially younger (mean 44.3 vs. 62.2 years, p < .001), consistent with a clinical pattern in which older patients present more often with isolated bony/degenerative stenosis while younger patients present more frequently with concurrent disc herniation. In the multivariable model adjusting for BMI and operative level, discectomy was not significant (exp [β] = 0.801, p = .112), and when age was added the discectomy coefficient moved further toward the null (exp [β] = 0.860, p = .330). The BMI and discectomy interaction was likewise non-significant (p = .203). Concurrent discectomy therefore carried no independent radiation signal after adjustment, and the univariable association is attributable to age confounding rather than the discectomy step itself.

## Limitations

Several limitations should be considered when interpreting these results. This is a retrospective, single-surgeon, single-institution study with a modest cohort size (n = 114). All cases were performed with a single C-arm and one imaging protocol, which means the data reported here may not transfer to centers with different equipment, workflow, or surgeon experience. The cohort reflects a surgeon with over 2,000 prior tubular decompression cases, so dose values should not be applied to surgeons in the learning curve, where dose per case runs higher. The primary outcome is C-arm reference air kerma, which captures the incident entrance-surface dose before backscatter is added (which may slightly underestimate the total skin dose received) and not deep-tissue absorbed dose [28]. Surgeon dose was not measured directly. Surgeon recommendations are inferred from machine released radiation dose rather than dosimetry badges. The upper-level subgroup is small (n = 7 at L2–L3 and n = 11 at L3–L4), widening the confidence interval around the upper-level effect. BMI values at the upper tail of the observed range (maximum 42 kg/m²) should be treated as extrapolations. BMI also serves as an imperfect proxy. The mechanistic driver is soft-tissue attenuation between the x-ray source and detector, which could be better captured with trunk anteroposterior diameter or pannus thickness. Radiation output may also vary with radiologic technologist practice, fluoroscopic projection and angulation, and the number of images acquired; these operator- and workflow-level factors were not captured and likely contribute to the share of dose variance the models did not explain. Although a priori calculation indicated adequate power for the primary BMI effect, the study was not adequately powered for the secondary upper versus lower level comparisons. The minimum detectable effect in the full multivariable model was approximately f^2^ = 0.07, so modest independent effects of age, sex, ASA class, laterality, or concurrent discectomy cannot be excluded. These secondary analyses should therefore be regarded as exploratory and hypothesis-generating.

## Conclusion

In this single-surgeon series of 114 consecutive tubular lumbar decompressions performed beyond the learning curve, BMI was the dominant determinant of intraoperative radiation. This effect operated through both longer fluoroscopy and greater output per second (consistent with automatic exposure control) rather than through increased surgical complexity or operative duration. Operative level was the only other significant predictor, with upper-level decompressions (L2–L3 and L3–L4) incurring approximately 50% greater dose than lower levels. However, the secondary analysis was underpowered to isolate whether L2–L3 or L3–L4 was the primary contributor to this effect, and larger cohorts are needed to confirm it. Across clinical scenarios, predicted Kar spanned roughly 7-fold, from 1.28 mGy (BMI 20, lower level) to 8.94 mGy (BMI 40, upper level). These findings provide a procedure-specific benchmark to guide preoperative planning and intraoperative stewardship in tubular spine surgery, with BMI serving as a preoperative flag for heightened dose-reduction measures.

## CRediT authorship contribution statement

**Conceptualization:** S Garg, P Young, A M Gallizzi, S S Gill. **Data curation:** S Garg, C Franquemont, P Young, P Parmaghante, J Lanni, N Garcia, B LaCarte. **Formal analysis:** S Garg, P Young, A M Gallizzi, S S Gill. **Investigation:** S Garg, C Franquemont, P Young, P Parmaghante, A K Gill, B LaCarte, E Piatt. **Methodology:** S Garg, P Young. **Project administration:** S Garg, P Young. **Software:** S Garg, C. Franquemont, A K Gill, E Piatt. **Supervision:** S Garg, P Young, A M Gallizzi, S S Gill. **Visualization:** S Garg. **Writing – original draft:** S Garg, C Franquemont, P Young, P Parmaghante, A K Gill, B LaCarte, E Piatt. **Writing—review and editing**: S Garg, C Franquemont, P Young, P Parmaghante, A K Gill, J Lanni, N Garcia, B LaCarte, E Piatt, A M Gallizzi, S S Gill.

## Funding

This research did not receive any specific grant from funding agencies in the public, commercial, or not-for-profit sectors.

## Declaration of competing interests

Dr Sanjitpal Gill declares potential financial interests or relationships as specified on required ICMJE-NASSJ Disclosure Forms. Dr Michael Gallizzi declares potential financial interests or relationships as specified on required ICMJE-NASSJ Disclosure Forms. All other authors declare that they have no known competing financial interests or personal relationships that could have appeared to influence the work reported in this paper.
